# Meta-analysis of the association between selenium and gastric cancer risk

**DOI:** 10.18632/oncotarget.7205

**Published:** 2016-02-05

**Authors:** He-Yi Gong, Jin-Guang He, Bao-Sheng Li

**Affiliations:** ^1^ Department of Radiation Oncology, Key Laboratory of Radiation Oncology of Shandong Province, Shandong Cancer Hospital and Institute, Jinan 250117, Shandong, P.R. China; ^2^ Department of Oncology, Heze Municiple Hospital, Heze 274031, Shandong, P.R. China; ^3^ Department of Radiation Oncology, Key Laboratory of Radiation Oncology of Shandong Province, Shandong Cancer Hospital and Institute, Jinan 250117, Shandong, P.R. China

**Keywords:** gastric cancer, selenium, mortality, association, meta-analysis

## Abstract

To clarify the effects of selenium level on the risk of gastric cancer (GC) and GC mortality, a meta-analysis was performed. Related studies were identified from PubMed, EMBASE, Springer Link, Ovid, Chinese Wanfang Data Knowledge Service Platform, Chinese National Knowledge Infrastructure (CNKI), and Chinese Biology Medicine (CBM). Pooled ORs and 95% CIs were used to assess the strengthof the associations. A total of 8 studies including 17834 subjects were involved in this meta-analysis. High selenium level was associated with GC risk in case-control study (OR = 0.62, 95% CI 0.44–0.89, *P* = 0.009; I^2^ = 52%) and cohort study (OR = 0.87, 95% CI 0.78–0.97, *P* = 0.01; I^2^ = 25%). In addition, high selenium level was associated with GC mortality risk (OR = 0.90, 95% CI 0.84–0.97, *P* = 0.006, I^2^ = 49%). In summary, this meta-analysis suggested that selenium might inversely associated with GC risk and GC mortality.

## INTRODUCTION

Gastric cancer (GC) represents a serious health problem on a global scale [1]. It is highly prevalent in Asia and is one of the leading causes of cancer-related death worldwide [1]. The current 5-year survival rate of individuals diagnosed with GC is about 24%, reflecting the reality that most cases are already in an advanced stage when diagnosed [2]. The pathogenic mechanisms underlying GC tumorigenesis remain unknown [3]. Therefore, predictive markers to identify high-risk population are urgentlyneeded for early detection and preventive care.

Ji et al. found that serum selenium level was 99.1 ± 31.8 ug/L in gastric cardia cancer (GCC) group and 121.8 ± 32.4 ug/L in gastric non-cardia cancer (GNCC) group (*P* = 0.044) [4]. Charalabopoulos and colleagues also suggested thatserum selenium levels were lower in the GC patient group than that in healthy individuals [5]. In addition, they found an inverse correlation between selenium and carcinoembryonic antigen (CEA) serum levels [5]. Therefore, the level of selenium might influence the risk of GC.

To date, several studies have investigated the association of selenium level with GC risk [6–13]. However, the results were inconsistent. Thus, we carried out a meta-analysis on the association of selenium level with GC risk. We also assessed the association between selenium level and GC mortality.

## RESULTS

### Eligible studies

**Figure 1:**
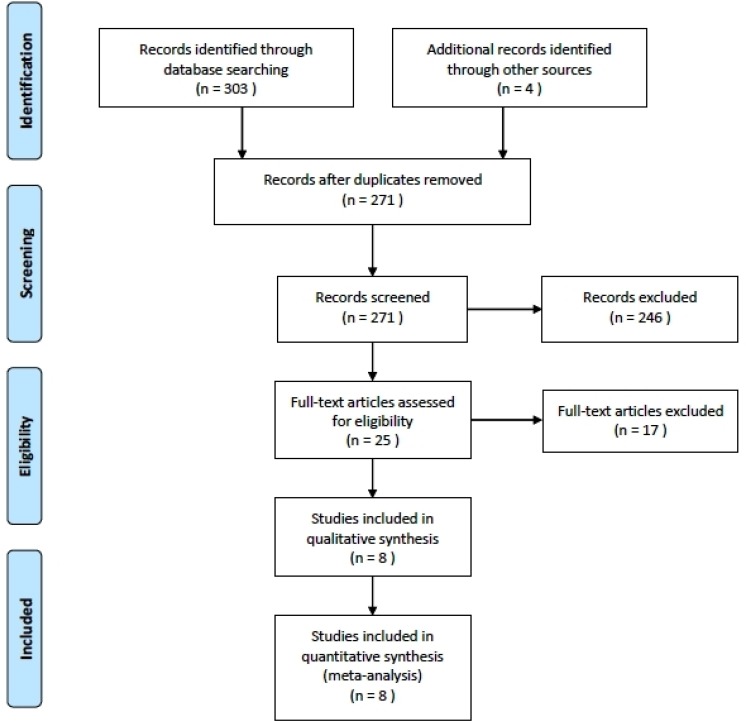
Flow chart of the study

In this current study, a total of 8 eligible studies with 17834 subjects met the inclusion criteria (Figure 1) [6–13]. The duration of follow-up ranged from 3 years to 17 years. Three articles reported two cohorts, and each cohort was considered as an independent study. There were 5 case-control studies and 3 cohort studies. There were 4 studies performed using Asians and 4 studies using Caucasians, respectively. Three studies collected the samples from toenails, while other collected from serum. Two studies reported the GC mortality risk. The characteristics of each study included in this meta-analysis are presented in Table 1.

**Table 1:** Characteristics of the included studies

First author	Year	Study	Race	Age	Men (%)	Follow-up	Sample	Sample	Cancer	Mortality	Covariant
		design				years	size	collection	location	reported	
Nomura	1987	Case-control	Asian	62.5	70	10	573	Serum	NA	NA	Age and serum cholesterol
Knekt 1	1990	Case-control	Caucasian	15–99	100	10	3037	Serum	NA	NA	Smoking, occupation, body mass index, parity, and cholesterol and hematocrit levels
Knekt 2	1990	Case-control	Caucasian	15–99	0	10	3037	Serum	NA	NA	Smoking, occupation, body mass index, parity, and cholesterol and hematocrit levels
van den Brandt	1993	Cohort	Caucasian	55–69	48	4	3500	Toenail	NA	NA	Age, gender, pack-years of past smokers, pack-years of current smokers, level of education, and intake of beta carotene and vitamin C
Kabuto	1994	Case-control	Asian	60	56	3	428	Serum	NA	NA	Age, sex, city, radiation dose, and smoking
Mark 1	2000	Case-control	Asian	57	60	5	1446	Serum	GCC	Yes	Age, sex
Mark 2	2000	Case-control	Asian	58	76	5	1149	Serum	GNCC	Yes	Age, sex
Wei 1	2004	Cohort	Asian	59	70	15	1103	Serum	GCC	Yes	Smoking, drinking, body mass index, and serum cholesterol
Wei 2	2004	Cohort	Asian	59	70	15	1103	Serum	GNCC	Yes	Smoking, drinking, body mass index, and serum cholesterol
Koriyama	2008	Case-control	Caucasian	NA	64	3	386	Toenail	NA	NA	Age, sex, hospital, season
Steevens	2010	Cohort	Caucasian	61	85	17	2072	Toenail	GCC	NA	Age, sex, cigarette smoking, number of cigarettes smoked daily, and numberof smoking years, alcohol consumption, BMI

GCC, gastric cardia cancer; GNCC, gastric noncardia cancer; BMI, body mass index; NA, not available

### Quantitative synthesis

The main results of this meta-analysis and the heterogeneity test were shown in Table 2. Selenium levelwas inversely associated with GC risk in case-control study (OR = 0.62, 95% CI 0.44–0.89, *P* = 0.009; I^2^ = 52%) and cohort study (OR = 0.87, 95% CI 0.78–0.97, *P* = 0.01; I^2^ = 25%). In the subgroup analysis by study design, a statistically significant association was found in case-control studies (OR = 0.61, 95% CI 0.40–0.93, *P* = 0.02) and in cohort studies (OR = 0.86, 95% CI 0.78–0.96, *P* = 0.006). In the subgroup analysis by race, GC risk was also found to be decreased in Asians (OR = 0.83, 95% CI 0.77–0.89, *P* < 0.00001) and Caucasians (OR = 0.53, 95% CI 0.32–0.88, *P* = 0.02). In the gender subgroup analysis, the inverse association between selenium level and GC risk was observed in women (OR = 0.82, 95% CI 0.74–0.90, *P* < 0.0001). A marginal association was observed in men (OR = 0.90, 95% CI 0.80–1.02, *P* = 0.09). In addition, the significant association was detected if the samples were collected from serum (OR = 0.82, 95% CI 0.76–0.88, *P* < 0.00001). Only a marginal association was found between toe nail selenium and risk of GC (OR = 0.65, 95% CI 0.40–1.07, *P* = 0.09). In the subgroup analysis by duration follow-up, selenium level was significantly associated with GC risk in the studies with less than 5 years follow-up (OR = 0.53, 95% CI 0.36–0.80, *P* = 0.002) and more than 5 years follow-up (OR = 0.84, 95% CI 0.79–0.89, *P* < 0.00001). Stratification by smoking status showed that non-smokers with high levels of selenium were associated with decreased GC risk (OR = 0.80, 95% CI 0.70–0.92, *P* = 0.001). No significant association was found between selenium level and GC risk in smokers (OR = 0.94, 95% CI 0.77–1.14, *P* = 0.53). The selenium level was also significant associated with decreased GC mortality risk (OR = 0.90, 95% CI 0.84–0.97, *P* = 0.006). In the subgroup analysis by cancer location, selenium level was significant associated with decreased GCC mortality risk (OR = 0.85, 95% CI 0.78–0.93, *P* = 0.0005). However, no significant association was found between selenium level and GNCC mortality risk (OR = 1.01, 95% CI 0.89–1.15, *P* = 0.84). Egger's test indicated no significant publication bias (*P* = 0.112 and *P* = 0.914).

**Table 2:** Results of this meta-analysis

	Association				Heterogeneity		
	OR (95% CI)	*Z*	*P* Value	Model	χ2	*P* Value	*I*^2^(%)
Gastric cancer risk							
Study design							
Case-control	0.62 (0.44–0.89)	2.61	0.009	R	12.44	0.05	52
Cohort	0.87 (0.78–0.97)	2.56	0.01	F	1.33	0.25	25
Race							
Asian	0.83 (0.77–0.89)	4.99	< 0.00001	F	1.67	0.64	0
Caucasian	0.53 (0.32–0.88)	2.43	0.02	R	13.01	0.01	69
Gender							
Male	0.90 (0.80–1.02)	1.70	0.09	R	9.07	0.03	67
Female	0.82 (0.74–0.90)	4.00	< 0.0001	F	2.40	0.49	0
Sample collection							
Serum	0.82 (0.76–0.88)	5.25	< 0.00001	F	8.50	0.13	41
Toenail	0.65 (0.40–1.07)	1.69	0.09	R	5.80	0.05	66
Duration follow-up							
Less than 5 years	0.53 (0.36–0.80)	3.08	0.002	F	1.20	0.55	0
More than 5 years	0.84 (0.79–0.89)	5.53	< 0.00001	F	8.68	0.12	42
Smoking status							
Smoker	0.94 (0.77–1.14)	0.62	0.53	R	8.41	0.01	76
Non-smoker	0.80 (0.70–0.92)	3.21	0.001	F	1.36	0.51	0
Gastric cancer mortality risk							
Overall	0.90 (0.84–0.97)	0.20	0.006	F	5.91	0.12	49
Location							
GCC	0.85 (0.78–0.93)	3.50	0.0005	F	1.27	0.26	21
GNCC	1.01 (0.89–1.15)	0.20	0.84	F	0.03	0.85	0

GCC, gastric cardia cancer; GNCC, gastric noncardia cancer; OR, odds ratio; CI, confidence interval; R, randomeffects model; F, fixed effects model.

## DISCUSSION

Although some studies analyzing the association between selenium level and GC, definite conclusions cannot be drawn. Therefore, we did this meta-analysis to estimate the relationship between selenium level and susceptibility to GC and mortality. We found that there was an inverse association between selenium level and GC risk. This result suggested that individuals with low selenium level might have increased GC risk or patients with GC may show shorter survival duration. In the subgroup analyses by study design, race, and duration follow-up, we found these factors did not influence the role of low selenium level in the development of GC. In the subgroup analyses by gender and sample collection, the marginal associations between low selenium level and GC risk were showed in men and toenail. Absence of ‘statistical significance’ does not rule out any etiologic link. The plausibility of a casual relation between antecedent selenium intake and cancer risk must be assessed according to different factors such as concordance between studies, dose-response relation and biological plausibility. Additionally, significant heterogeneity was found in these two subgroup analyses and may influence the results. Thus, more studies are needed to assess the associations between low selenium level and GC risk in men and the studies with toenail collection. Smoking was a risk factor of GC. In this meta-analysis, we found that selenium did not show protective role of GC in smokers. As for GC mortality risk, GC patients with high seleniumlevel might have low mortality risk. However, this effect was only observed in GCC but not in GNCC. Previous studies suggested that serum selenium level was highly correlated with the location of GC [4]. However, the underlying mechanism was still unknown. This issue should be investigated in the future.

Evidence indicated several mechanisms for selenium anticarcinogenesis: altered carcinogen metabolism, cell cycle regulation, immune surveillance, cell death programming, cancer cell migration and angiogenesis [14, 15]. Animal studies suggested that supplementation with vitamins and with selenium yielded *H. pylori* recovery from 17% of challenged animals, compared with 43% of those fed a control diet [16]. Thus, selenium supplements might prevent GC or GC mortality. To determine the long-term effect of vitamin E and selenium on risk of prostate cancer in relatively healthy men, Klein and colleagues performed the Selenium and Vitamin E Cancer Prevention Trial (SELECT) [17, 18]. Compared with placebo, the absolute increase in risk of prostate cancer per 1000 person-years was 1.6 for vitamin E, 0.8 for selenium, and 0.4 for the combination [18]. However, in a recentCochrane review, the investigators suggested that the effects of selenium supplementation on cancer risk were inconsistent and no convincing evidence suggested that selenium supplements can prevent cancer in humans [19].

We had to mention the importance of heterogeneity and publication bias, which might influence the results of meta-analysis. In our study, significant heterogeneity was observed. We used subgroup analysis to explore the sources of heterogeneity. We found that *I*^2^ value was decreased in the subgroup by the duration of follow-up. The result suggested that duration of follow-up might be the major source of the heterogeneity. However, heterogeneity did not seem to influence the results, because the significance of the result was not altered in this subgroup. Additionally, Egger's tests were used to find potential publication bias. The results indicated that there was no significant publication bias.

In summary, this meta-analysis suggested that selenium levels were inversely associated with the risk of GC risk and GC mortality. Large and well-designed studies are warranted to validate our findings.

## MATERIALS AND METHODS

### Publication search

We searched databases containing PubMed, EMBASE, Springer Link, Ovid, Chinese Wanfang Data Knowledge Service Platform, Chinese National Knowledge Infrastructure (CNKI), and Chinese Biology Medicine (CBM) up to 10 Apr 2015, using the following Mesh terms: (“Gastric Neoplasms” [MeSH] or “gastric cancer” or “stomach tumor” or “stomach carcinoma” or “carcinoma of stomach”) and (“selenium” or “Se”). The references from retrieved articles were alsosearched. There was no limit set in the searches.

### Inclusion criteria and data extraction

Studies included in this meta-analysis have to meet the following criteria: (1) case–control study or cohort study studying on association between selenium and risk of GC or GC mortality; (2) all patients with the diagnosis of GC confirmed by pathological or histological examination; (3) sufficient published data about sample size, odds ratio (OR), and their 95% confidence interval (CI). Studies wereexcluded when they were: (1) not case–control study orcohort study; (2) duplicate of previous publication; (3) based on incomplete data; (4) meta-analyses, letters, reviews, or editorial articles.

Data were independently extracted by two reviewers using a standardized data extraction form. Discrepancies were resolved by discussion and if consensus was not achieved the decision was made by all the reviewers. The title and abstract of all potentially relevant articles were screened to determine their relevance. Full articles were also scrutinized if the title and abstract were ambiguous. The following information was collected from each study: authors, year of publication, study design, race, age, sex, years of follow-up, sample size, sample collection, GC location, and covariant.

### Statistical analysis

Statistical analysis was conducted by using STATA statistical package (version 11, STATA, College Station, TX). The association of selenium leveland GC risk or GC mortality was estimated by OR with 95% CI. The heterogeneity was tested by the Q-statistics with *P*-values < 0.1. Dependent on the results of heterogeneity test among individual studies, the fixed effect model (Mantel–Haenszel) or random effect model (DerSimonian and Laird) was selected to summarize the combined OR and their 95% CI. The significance of the pooled OR was determined by the *Z* test. Subgroup analyses were carried out bystudy design, race, gender, smoking status, years of follow-up, sample collection, and cancer location, if possible. Publication bias was investigated with Egger's linear regression test. All the *P* values were two sided. *P* value less than 0.05 was considered statistically significant.
